# Tuneable pressure effects in graphene oxide layers

**DOI:** 10.1038/s41598-017-12444-x

**Published:** 2017-09-22

**Authors:** Yusuke Sekimoto, Ryo Ohtani, Masaaki Nakamura, Michio Koinuma, Leonard F. Lindoy, Shinya Hayami

**Affiliations:** 1 Department of Chemistry, Graduate School of Science and Technology, Kumamoto University, 2-39-1 Kurokami, Chuo-ku, Kumamoto, 860-8555 Japan; 2 School of Chemistry, The University of Sydney, Sydney, NSW 2006 Australia; 3 Institute of Pulsed Power Science (IPPS), Kumamoto University, 2-39-1 Kurokami, Chuo-ku, Kumamoto, 860-8555 Japan

## Abstract

Tuneable pressure effects associated with changing interlayer distances in two-dimensional graphene oxide (GO)/reduced GO (rGO) layers are demonstrated through monitoring the changes in the spin-crossover (SCO) temperature (*T*_1/2_) of [Fe(Htrz)_2_(trz)](BF_4_) nanoparticles (NPs) incorporated in the interlayer spaces of the GO/rGO layers. The interlayer separation along the GO to GO/rGO-NP composites to rGO series decreases smoothly from 9.00 Å (for GO) to 3.50 Å (for rGO) as the temperature employed for the thermal reduction treatments of the GO-NP composites is increased. At the same time, *T*_1/2_ increases from 351 K to 362 K along the series. This *T*_1/2_ increment of 11 K corresponds to that observed for pristine [Fe(Htrz)_2_(trz)](BF_4_) NPs under a hydrostatic pressure of 38 MPa. The influence of the stacked layer structures on the pseudo-pressure effects has been further probed by investigating the differences in *T*_1/2_ for [Fe(Htrz)_2_(trz)](BF_4_) that is present in the composite as larger bulk particles rather than as NPs.

## Introduction

Van der Waals interactions in the pores of micro-porous materials are known to generate a confinement effect for guest species^1–4^. Confinement effects, in particular pseudo-pressure effects, are of importance to the development of characteristic states and unique phases of materials in pores under mild conditions. For example, single-walled carbon nano-horns produce a pseudo-pressure effect corresponding to *ca*. 1.9 GPa on KI nanocrystals in the pores, giving rise to a structural transition to a high pressure KI phase under ambient pressure^5^. Similar effects have been demonstrated for gas molecules accommodated in metal-organic frameworks. O_2_ molecules behave in a similar manner to their solid state above the freezing point of O_2_ in nano-channels of [{[Cu_2_(pzdc)_2_(pyz)]·2H_2_O}_n_] (pzdc = pyrazine-2,3-dicarboxylate), with the simultaneous formation of (O_2_)_2_ dimers having also been reported^6^. Recently, stacking structures of two-dimensional (2D) nano-sheets of graphene have been shown to act as a ‘field’ that produces similar effects to confinement effects^7–9^. For example, in highly ordered pyrolytic graphite (HOPG), mixtures of water and methanol form a stable two-dimensional (2D) molecular assembly that is similar to a transient assembly that occurs in the bulk liquid^7^. In the graphene sheet case, an extended H-bond network forms between water and methanol, also giving rise to the formation of such a stable 2D structure. In these cases, the relationship between the resulting pressure (*P*) and the interlayer distance (*d*) is given by *P* ≈ *E*_*w*_/*d*, where *E*_*w*_ is the adhesion energy^8–10^. Although there are many types of porous materials, tuneable pressure effects arising from pore size tuning within a single (composite) material remains largely unexplored. In most cases, materials with different size pores need to be prepared one by one to obtain desired pseudo-pressure effects^11,12^. Clearly materials that are capable of exhibiting tuneable pressure effects would potentially provide a useful means for precisely controlling particular physical properties of nanomaterials confined in the pores. In this study, we have focused on the structural transformation from graphene oxide (GO) to reduced graphene oxide (rGO) for the development of such tuneable pressure effects.

**Figure 1:**
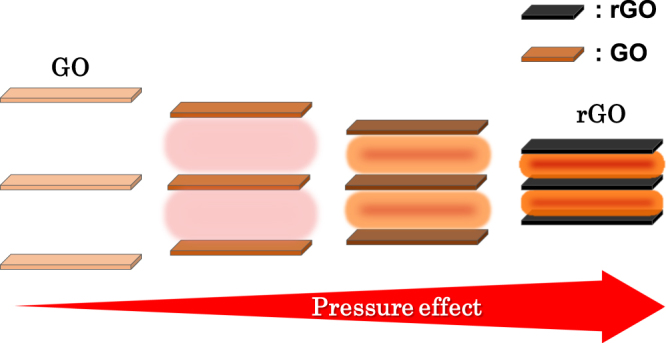
Schematic illustration of the tuneable pressure effects caused by the transformation of GO to rGO.

GO, an oxidation product of graphene, consists of 2D layers incorporating oxygen functional groups such as epoxy, hydroxyl and carboxyl groups. Our group has demonstrated its high proton conductivity^13–15^ and developed composites with cationic species that include metal ions and metal complexes^16,17^. Oxygen functional groups on GO are removed by thermal treatment, yielding rGO. At the same time, the interlayer distances decrease progressively from 7–9 Å in GO to 3–4 Å in rGO^18–21^. Importantly, interlayer distances are influenced by the quantity of functional groups on the layers which, in turn, can be controlled by changing the temperature employed for the thermal treatment. In the present study, we anticipated that the above structural transformation between GO and rGO would lead to tuneable pressure effects that reflect changes in the respective interlayer distances (Fig. 1).

In order to demonstrate the presence of pressure effects in GO/rGO layer structures, we have focused on spin-crossover (SCO) phenomena that occurs for [Fe(Htrz)_2_(trz)](BF_4_) nanoparticles (NPs) confined in the interlayer spaces. Iron(II) complexes show SCO phenomena in which electron configurations are switched between high-spin (HS) and low-spin (LS) states with thermal hysteresis^22–24^. SCO temperatures (*T*_1/2_) have been shown to be sensitive to the presence of a hydrostatic pressure that acts to restrict a structural transformation synchronized with the SCO. A correlation between *T*_1/2_ and pseudo-pressures for various compounds has been reported^25–27^. In a prior study Colacio and co-workers have described a *T*_1/2_-pressure correlation for [Fe(Htrz)_2_(trz)](BF_4_) NPs^27^. Thus, it appeared feasible that we could estimate pseudo-pressure values arising from the confinement effect using [Fe(Htrz)_2_(trz)](BF_4_) NPs confined within GO/rGO layers by investigating changes in *T*_1/2_, with the latter changes mirroring changes in the corresponding interlayer distances.

In the present study, we demonstrate that pseudo-pressure effects, generated by transformation of GO to rGO, leads to changes in *T*_1/2_ for [Fe(Htrz)_2_(trz)](BF_4_) NPs that are accommodated between the GO/rGO layers. *T*_1/2_ shows a smooth increase as the interlayer distance is decreased, thus demonstrating the presence of tuneable pressure effects in the GO/rGO layers.

## Results

### GO/rGO-[Fe(Htrz)_2_(trz)](BF_4_) NP composites

The [Fe(Htrz)_2_(trz)](BF_4_) NPs were prepared using a ligand-melt method from FeCl_2_·4H_2_O, 1-H-1,2,4-triazole and NaBF_4_ (see Methods)^23^. Their size was 32 ± 17 nm, as determined from their scanning electron microscopy (SEM) and transmission electron microscopy (TEM) images (Supplementary Figure 1a and b). These NPs showed SCO behaviour at 351 K with a thermal hysteresis of 23 K (Supplementary Figure 2).

**Figure 2:**
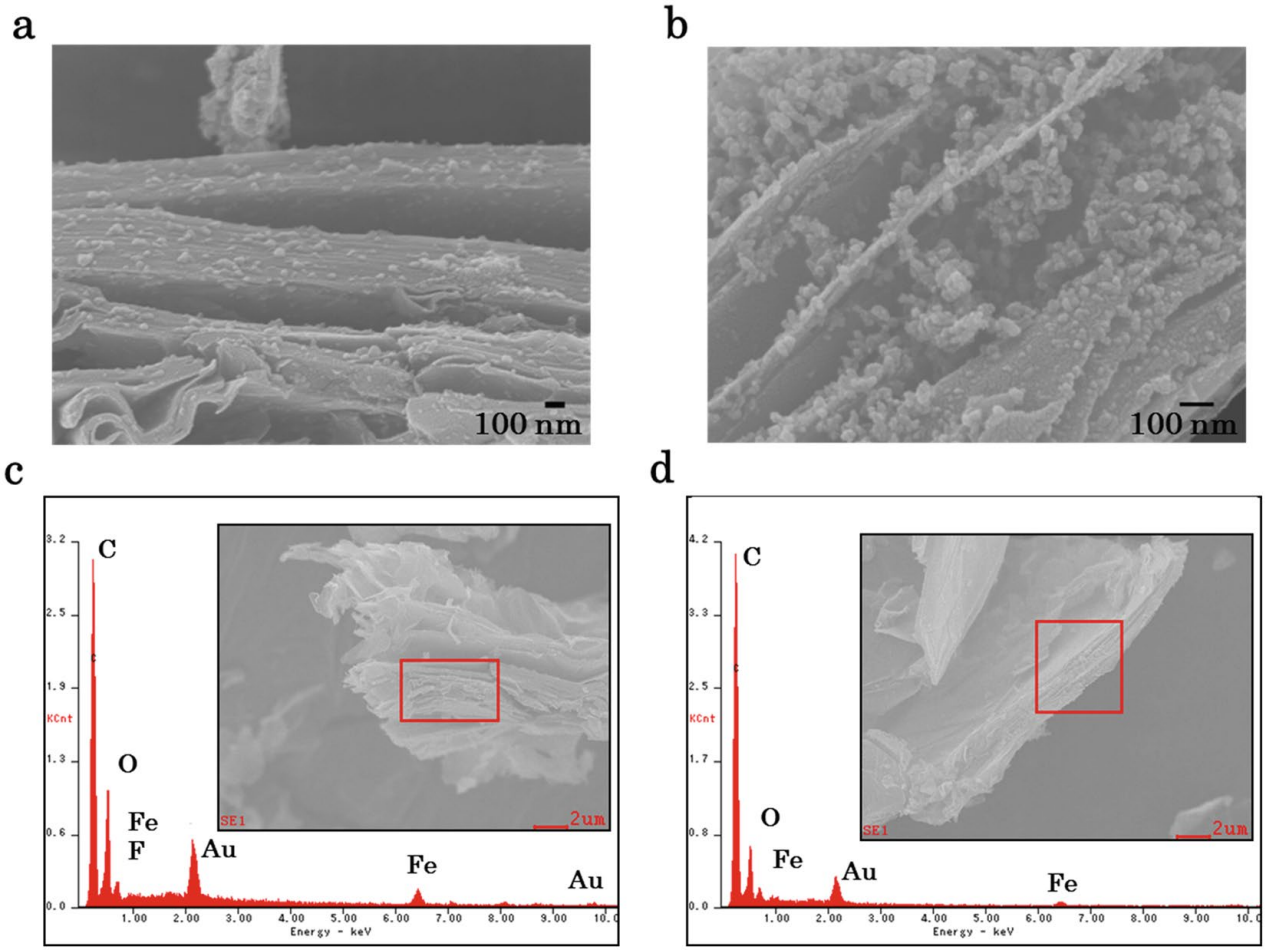
SEM images of (**a**) **1** and (**b**) **4**. SEM-EDX results for (**c**) **1** and (**d**) **4**. Squares indicate areas analysed by EDX spectroscopy. Peaks for Au are caused by sputtering treatments with Au.

The GO composite (**1**) incorporating [Fe(Htrz)_2_(trz)](BF_4_) NPs was prepared by mixing GO and [Fe(Htrz)_2_(trz)](BF_4_) NPs in a mass ratio of 1:2 in ethanol followed by filtration. Subsequently, thermal reduction treatments at 373 K, 423 K, and 473 K were carried out on **1**, resulting in the formation of the reduced GO (rGO) composites **2**, **3**, and **4**, respectively. The transformations from GO to rGO in these composites were corroborated by investigating their current-voltage (IV) properties, X-ray photo-electron spectroscopy (XPS) spectra, thermogravimetric (TG) behaviour and Raman spectra (Supplementary Figures 3, 4, 5 and 6). SEM images and SEM-energy dispersive X-ray spectroscopy results for pristine GO, **1** and **4** confirmed that the NPs were incorporated in the interlayer spaces in these latter materials (Fig. 2 and Supplementary Figure 7).

In order to probe the composition and electronic states of the [Fe(Htrz)_2_(trz)](BF_4_) NPs in the composites, **1** and **4**, these products were further characterized using Fourier transform infrared spectroscopy (FT-IR) and XPS. Although the IR spectrum of **4** exhibits small peaks, the peak positions correspond to those for the [Fe(Htrz)_2_(trz)](BF_4_) NPs^28^, in agreement with the structure of [Fe(Htrz)_2_(trz)](BF_4_) remaining intact in the composite **4** generated by the thermal treatment of **1** at 473 K (Supplementary Figure 8). This is also confirmed by the PXRD results as described in the next paragraph. The peaks for Fe2p_3/2_ in the XPS spectra of **1** and **4**, are present at 711.2 eV; that is, at a higher energy than the 709.0 eV observed for [Fe(Htrz)_2_(trz)](BF_4_) NPs (Supplementary Figure 4c). This peak shift of 2.2 eV is in accord with electronic interactions occurring between the incorporated [Fe(Htrz)_2_(trz)](BF_4_) NPs and the GO/rGO surfaces.

**Figure 3:**
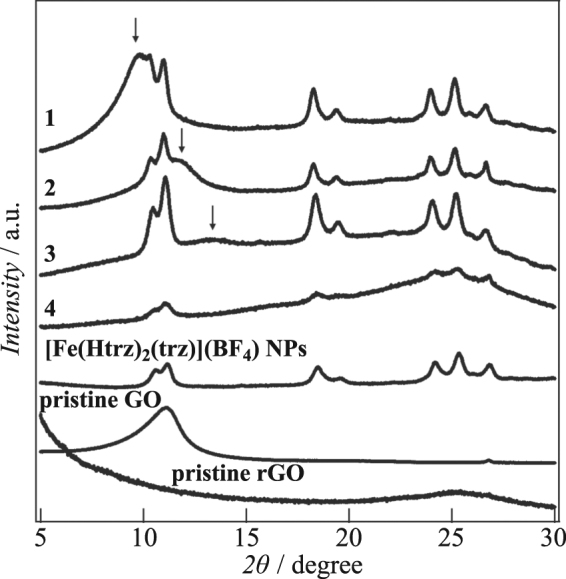
PXRD patterns for **1–4**, [Fe(Htrz)_2_(trz)](BF_4_) NPs, pristine GO and pristine rGO.

The interlayer distances in the stacked structures of **1–4** were investigated by powder X-ray diffraction (PXRD) (Fig. 3 and Table 1). Each of the patterns exhibit a broad peak arising from the stacked layer structures as well as peaks for the [Fe(Htrz)_2_(trz)](BF_4_) NPs.

**Table 1:** Interlayer distances and SCO temperatures for 1–4.

	Interlayer distance (Å)	*T*_1/2_ (K)	*T*_1/2_^↑^ (K)	*T*_1/2_^↓^ (K)	Δ*T* (K)
**1**	9.00	351	358	343	15
**2**	7.45	355	366	343	23
**3**	6.77	358	368	348	20
**4**	3.50–3.78	362	374	349	25

The interlayer distance in **1** is 9.00 Å (2*θ* = 9.82°) which is larger than that of pristine GO, 7.92 Å (2*θ* = 11.16°), in accord with the accommodation of NPs between layers (see Fig. 6). Following thermal treatments of **1**, the interlayer distances decrease because of the removal of the oxygen functional groups from the GO layers. The distances are 7.45 Å (2*θ* = 11.86°) for **2** and 6.77 Å (2*θ* = 13.07°) for **3**, clearly showing that the interlayer distances decrease as the thermal treatment temperature is raised. In the case of **4** (which was treated at 473 K), a broad peak occurred around 2*θ* = 25° that corresponds to that for pristine rGO (prepared by thermal treatment of pristine GO at 473 K) (Fig. 3). From this result, we concluded that the interlayer separation in **4** is 3.50–3.78 Å (2*θ* = 23.5–25.5°). The decrease of peak intensities of NPs was observed in **4**, which is caused by likely the loss of crystallinity of NPs in layers by the thermal treatment.

**Figure 4:**
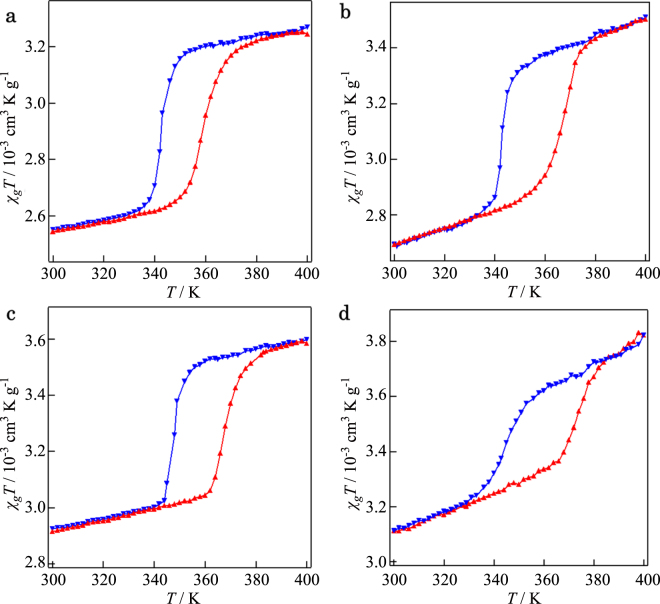
SCO behaviour of (**a**) **1**, (**b**) **2**, (c) **3** and (**d**) **4**. Heating: (), cooling: ().

**Figure 5:**
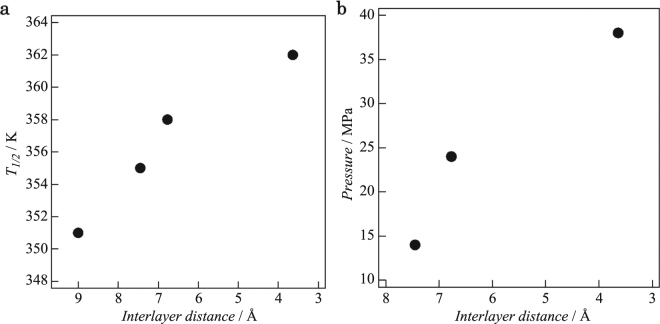
Correlations (**a**) between the interlayer distances and *T*_1/2_ for **1–4**, and (**b**) between the interlayer distances and the estimated pseudo-pressure values for **2–4**.

The effect of differences in the interlayer distances on the SCO behaviour of the NPs was investigated using magnetic susceptibility measurements for **1–4** employing a SQUID magnetometer in the temperature range of 300–400 K (Fig. 4). Initially we carried out the measurements from 400 K to 300 K and then subsequently from 300 K to 400 K to avoid the possibility of solvent effects affecting the magnetic results. Although the *χ*_*m*_ *T* values were not estimated accurately because of the difficulty in determining the quantities of [Fe(Htrz)_2_(trz)](BF_4_) NPs present in the respective GO/rGO layers, each of **1–4** exhibited SCO behaviour with thermal hysteresis occurring at different temperatures. Thus, **1** showed SCO at 351 K with a *T*_1/2_ value corresponding to that for pristine [Fe(Htrz)_2_(trz)](BF_4_) NPs. After thermal treatment, *T*_1/2_ increased to 355 K for **2**, 358 K for **3**, and 362 K for **4**, respectively. These results reveal a correlation between *T*_1/2_ and the interlayer distances in **1–4**, with *T*_1/2_ increasing as the interlayer distances decrease (Fig. 5a and Table 1). Since compositional and structural changes for the [Fe(Htrz)_2_(trz)](BF_4_) NPs do not occur in **1–4**, we conclude that the above correlation demonstrates the presence of pseudo-pressure effects that are reflected by the changes in *T*_1/2_ for the respective NP composites. With respect to this, Colacio and co-workers have reported that the [Fe(Htrz)_2_(trz)](BF_4_) NPs show a linear dependency of *T*_1/2_ on ‘bulk-scale’ hydrostatic pressures (*p*) given by *T*_1/2_(*p*) = *T*_1/2 + _290(66)*p* ^27^. We applied this relationship to our results for **1–4** in order to estimate pseudo-pressure values for these systems: producing 14 MPa, 24 MPa and 38 MPa for **2**, **3** and **4**, respectively (Fig. 5b). Although the effects on the hysteresis widths (Δ*T*) associated with cooperativity in **1–4** remain unclear at this stage, they are anticipated to be less than those for *T*_1/2_ (Table 1).

### GO/rGO**-**[Fe(Htrz)_2_(trz)](BF_4_) larger particle composites

**Figure 6:**
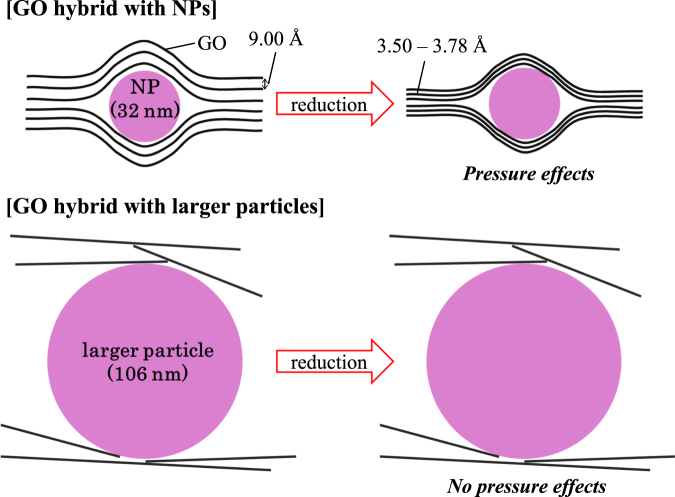
Schematic representations of the confinement effects in GO composites with NP and bulk [Fe(Htrz)_2_(trz)](BF_4_).

In an extension of the study aimed at probing the particle size dependency as well as the contribution of the respective layered structures to the pseudo–pressure effects discussed above, larger particles of [Fe(Htrz)_2_(trz)](BF_4_) with a size of 106 ± 25 nm (*T*_1/2_ = 357 K; Supplementary Figure 9) were prepared along with their GO and rGO composites, **5** and **6** ^24^. The latter were synthesized using the same procedures as used to obtain **1** and **4** respectively (see Methods). In contrast to the PXRD patterns for **1–4**, those for **5** and **6** exhibit no peaks that can be assigned as arising from the presence of a 2D stacking structure. This is in accord with the GO/rGO layers no longer being stacked regularly due to the [Fe(Htrz)_2_(trz)](BF_4_) particles being too large to be accommodated in the interlayer spaces, resulting in mixtures of [Fe(Htrz)_2_(trz)](BF_4_) particles and GO or rGO being formed (Fig. 6 and Supplementary Figure 10). The XPS spectra of **5** and **6** show peaks for Fe2p_3/2_ at 708.9 eV, corresponding to the value observed for pristine [Fe(Htrz)_2_(trz)](BF_4_) particles (Supplementary Figure 4d). Therefore, in contrast to the NP composites, electronic interactions between the [Fe(Htrz)_2_(trz)](BF_4_) particles and GO/rGO layers are not evident for **5** and **6**. Importantly, *T*_1/2_ values for **5** and **6** are 357 K and 352 K, respectively, demonstrating that an increase in *T*_1/2_ does not occur from **5** to **6** - again contrasting with the behaviour of the corresponding NP composites (Supplementary Figure 11). From these results, we conclude that the ordered stacking structures and the small size (and larger collective surface area) of the NPs are necessary to develop the interactions leading to the interlayer pseudo-pressure effects discussed in this manuscript (Fig. 6).

## Discussion

We have demonstrated the presence of tuneable pressure effects generated in the interlayers of GO and rGO materials for the first time by monitoring the changes in the SCO temperatures (*T*_1/2_) of [Fe(Htrz)_2_(trz)](BF_4_) NPs occupying the interlayer spaces. Shorter interlayer distances lead to larger pseudo-pressure effects on *T*_1/2_. The interlayer distances can be adjusted by varying the thermal treatment temperatures used to obtain the GO/rGO composites. In regular rGO, a pseudo-pressure value of 38 MPa was obtained corresponding to an adhesion energy of 0.86 meV Å^−2^ ^29,30^. Our findings provide insight towards the application of GO materials exhibiting tuneable pressure effects for provision of reaction fields for molecular conversions as well as for the development of new composites incorporating functional nano-materials such as quantum dots, metal complexes or even 2D nanosheets.

## Methods

### Syntheses

All chemicals and solvents were purchased from commercial sources and used without further purification.

[Fe(Htrz)_2_(trz)](BF_4_) nanoparticles were synthesized by the previously described procedure of Bousseksou *et al*.^23^. FeCl_2_·4H_2_O (200 mg), NaBF_4_ (110 mg) and 1-H-1, 2, 4-triazole (5 g) were mixed without solvent and the mixture immediately heated at 423 K. After stirring for 1 min, the resulting melt was left to cool to room temperature. The crude red purple crystalline material obtained was dispersed in ethanol (50 mL) and the mixture centrifuged. The solid was collected by filtration using a membrane filter (1 μm) and the red purple product obtained was dried under vacuum at room temperature.

Graphene oxide was prepared by a modification of Hummer’s method^15^. Graphite (2 g), grated NaNO_3_ (2 g) and H_2_SO_4_ (90 mL) were mixed in a round-bottom flask. The mixture was cooled in an ice bath for 30 min with stirring. Powdered KMnO_4_ was added slowly to the flask. The resultant mixture was stirred at 308 K for 30 min. Distilled water (180 mL) was added very slowly and the mixture was stirred at 368 K for 1 h. Then, 30% H_2_O_2_ solution (30 mL) was added very carefully. Finally, distilled water (800 mL) was added to quench the reaction. The mixture was centrifuged at 3000 rpm and the supernatant liquid was removed. The resulting solid was washed with 5% HCl once and then with distilled water three times then dried at 313 K. It was then dispersed in ethanol (0.1 g/150 mL) using ultrasonication for 2 h. This solution was centrifuged at 4000 rpm for 1 h; the supernatant liquid consisted of the graphene oxide (GO) dispersion.

GO composite (**1**) was prepared by mixing GO/ethanol dispersion (0.1 g/150 mL) with [Fe(Htrz)_2_(trz)](BF_4_)/ethanol dispersion (0.2 g/150 mL) and stirring the mixture at room temperature for 6 h. The black product was centrifuged and the solid collected by a membrane filter (1 μm), washed with ethanol, and dried under vacuum at room temperature.

rGO composites were prepared by thermal reduction treatments of **1**. Annealing of **1** at 373 K, 423 K, and 473 K was carried out in a vacuum for 12 h and resulted in the formation of the respective rGO composites **2**, **3**, and **4**.

Bulk crystalline [Fe(Htrz)_2_(trz)](BF_4_) was prepared by the method described previously^24^. A solution of 1-H-1, 2, 4-triazole (2.09 g) in 10 mL of ethanol and a solution of NaBF_4_ (2.20 g) in 20 mL of water were mixed under an Ar atmosphere. FeCl_2_·4H_2_O (2.00 g) was added to the solution under Ar, and the reaction mixture was stirred for 24 h. The solution was then centrifuged at 4000 rpm for 1 h. The precipitate was isolated by filtration and washed with ethanol. The pink product was dried in a vacuum at room temperature.

GO composite incorporating bulk [Fe(Htrz)_2_(trz)](BF_4_) (**5**) and the corresponding rGO composite (**6**) were prepared by the same procedure as used to obtain **1** and **4**, respectively. **5** was prepared by mixing GO/ethanol dispersion (0.1 g/150 mL) with bulk [Fe(Htrz)_2_(trz)](BF_4_)/ethanol dispersion (0.2 g/150 mL) and stirring the mixture at room temperature for 6 h. The black product was centrifuged and the precipitate collected by a membrane filter (1 μm), washed with ethanol and dried under vacuum at room temperature. **6** was prepared by a thermal reduction treatment of **5** at 473 K.

### Measurements

Scanning electron microscopy (SEM) and SEM-energy dispersive X-ray spectroscopy (EDX) were carried out on a JEOL, JSM-7600 F instrument. Transmission electron microscopy (TEM) was carried out on a JEOL, 2000FX, 200 kV electron microscope. Magnetic susceptibilities were measured on a superconducting quantum interference device (SQUID) magnetometer, Quantum Design, MPMSXL-5. IV properties were measured using a electrochemical analyzer, BAS, Model ALS/DY2323 BI-POTENTIOSTAT. The temperature dependence of the electrical resistivity was measured by means of a Keithley, 2182 A Digital Nanovoltmeter. X-ray photo-electron spectroscopy was carried out a Thermo Scientific, ThetaProbe Angle-Resolved X-ray Photoelectron Spectrometer System. Thermogravimetric analysis (TGA) was carried out on a SEIKO, EXSTAR TG/DTA 6300 thermogravimetric analyzer. Micro Raman spectroscopy was performed on a Jasco, NRS-3100 spectrometer, with a 532 nm excitation source. Fourier transform infrared spectroscopy was performed on a PerkinElmer, Spectrum Two spectrometer. Powder X-ray diffraction (PXRD) patterns were obtained on a Rigaku, MiniFlex II X-ray diffractometer.

## Electronic supplementary material

Tuneable pressure effects in graphene oxide layers
