# Supplementary material for: Tuneable pressure effects in graphene oxide layers
Source: Sci Rep. 2017 Sep 22;7:12159. doi: 10.1038/s41598-017-12444-x (PMC5610342; doi:10.1038/s41598-017-12444-x)
Supplement: Supplementary file 1 — Tuneable pressure effects in graphene oxide layers [file 41598_2017_12444_MOESM1_ESM.pdf]

Supplementary Information

Tuneable pressure effects in graphene oxide layers

Yusuke Sekimoto, Ryo Ohtani, Masaaki Nakamura, Michio Koinuma, Leonard F. Lindoy, and Shinya Hayami

Corresponding author: S. Hayami, hayami@kumamoto-u.ac.jp

SUPPLEMENTARY FIGURES

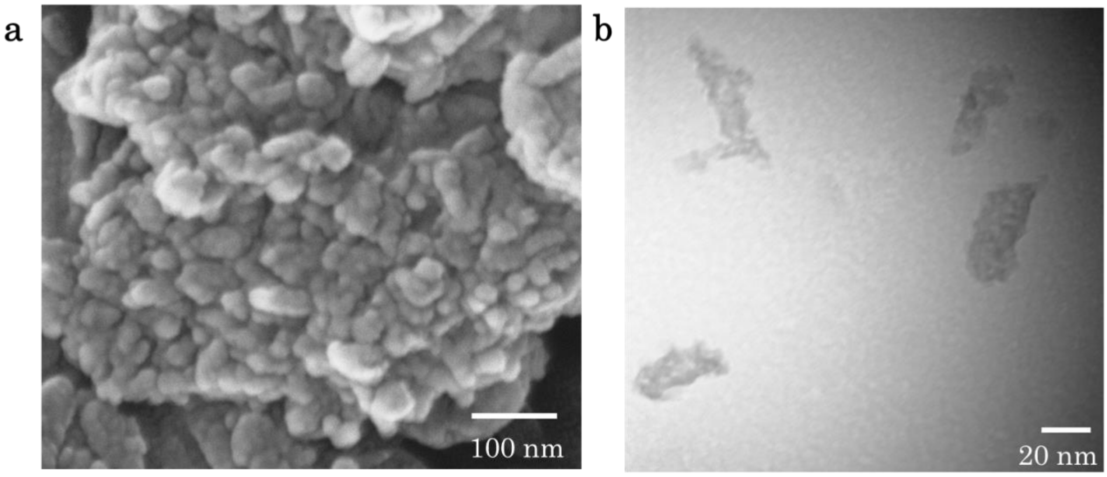

Supplementary Figure 1 (a) SEM image and (b) TEM image of $[Fe(Htrz)_2(trz)](BF_4)$ NPs.

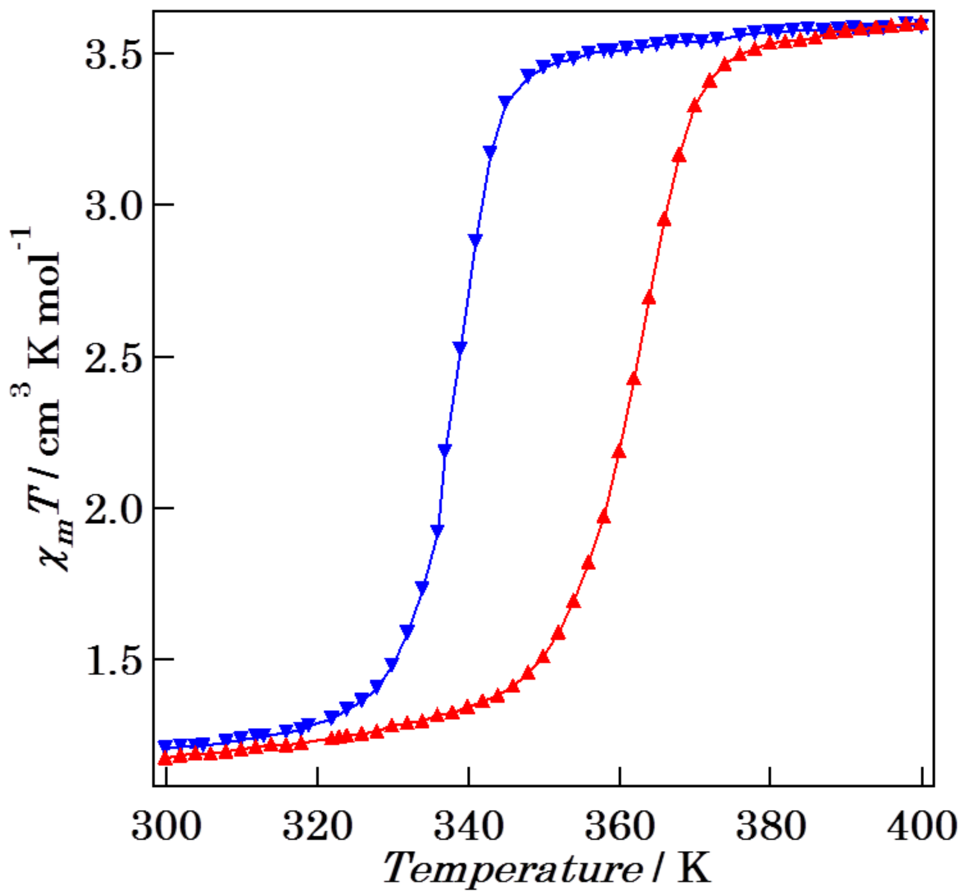

Supplementary Figure 2 The thermal dependence of the $\chi_m T$ value for the $[Fe(Htrz)_2(trz)](BF_4)$ NPs; heating mode (▲) and cooling mode(▼).

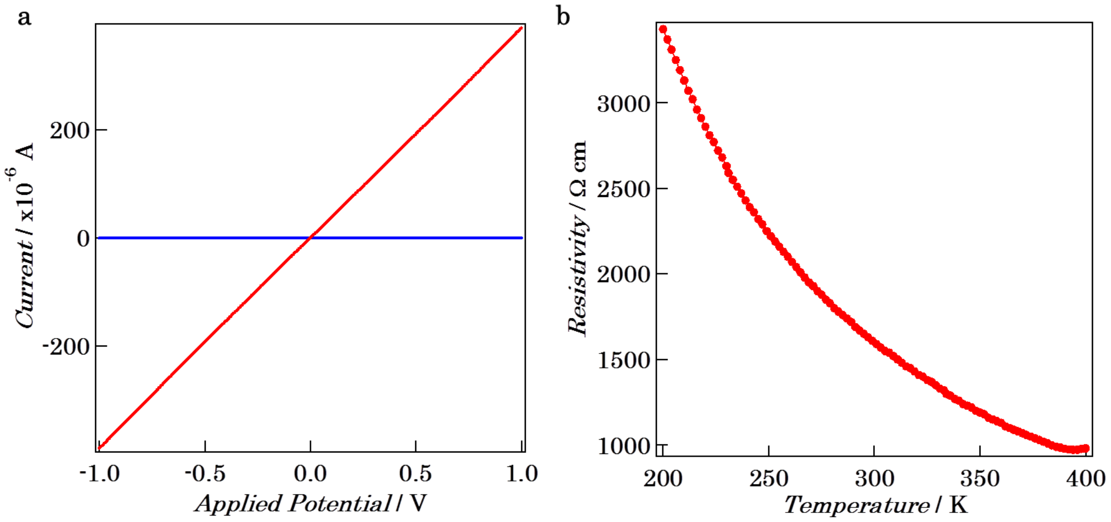

Supplementary Figure 3 (a) IV curves for **1** (blue) and **4** (red). (b) The temperature dependence of resistivity for **4**. For IV curves, **1** shows the insulator property in accord with the behaviour of GO. Electron conductivity of **1** was 2.51×10^{-10} A at applied 1 V. On the other hand, **4** showed 3.89×10^{-4} A at 1 V, in accord with the oxygen functional groups being removed to yield rGO. The resistivity of **4** is increased with decreasing temperature as shown in (b). This result shows that **4** is a semiconductor.

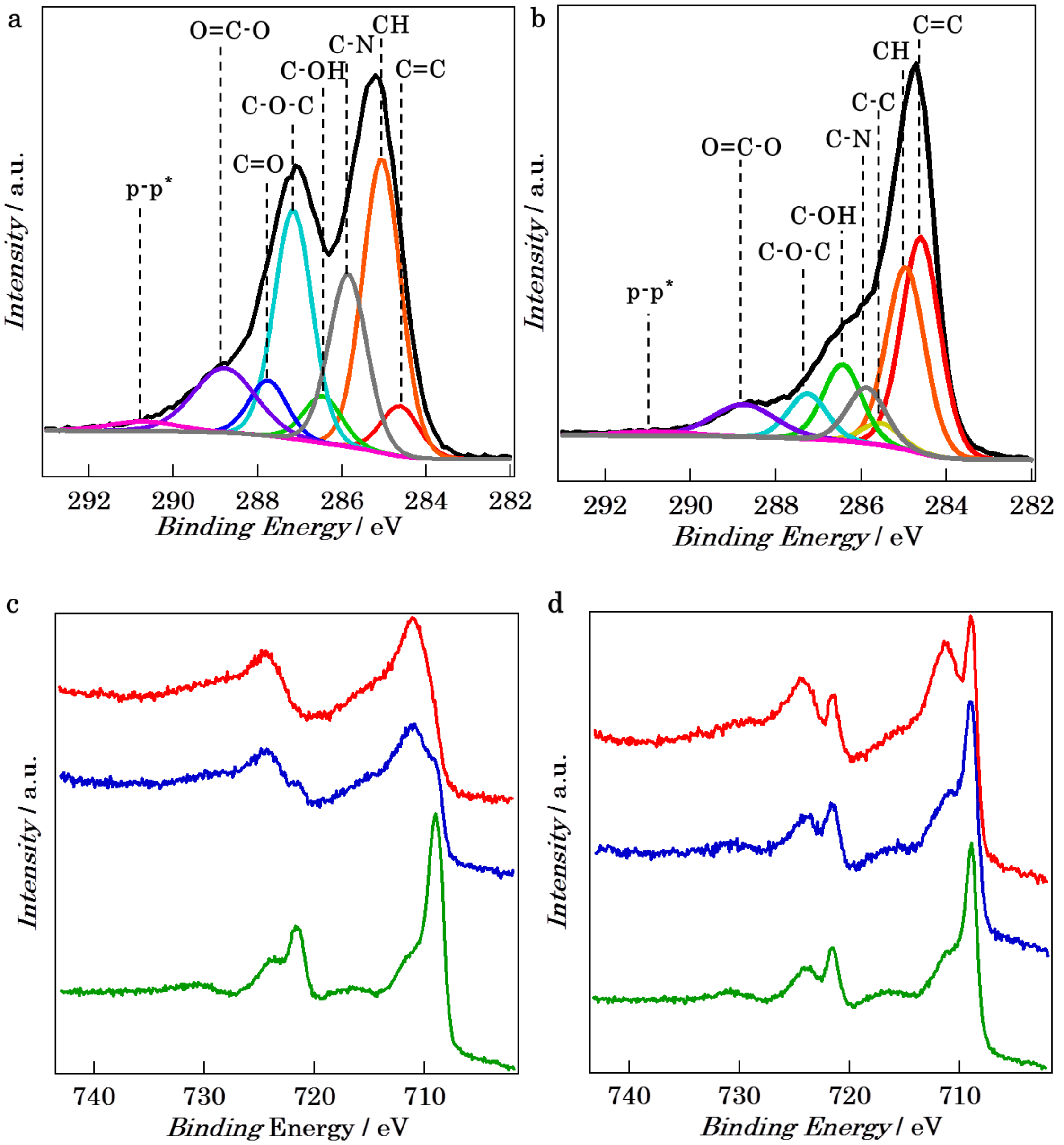

Supplementary Figure 4 X–ray photo–electron spectroscopy (XPS) spectra of C1s for (a) **1** and (b) **4**. (c) XPS spectra of Fe2p for [Fe(Htrz)$_2$(trz)](BF$_4$) NPs (green), **1** (blue) and **4** (red). (d) XPS spectra of Fe2p for bulk [Fe(Htrz)$_2$(trz)](BF$_4$) (green), **5** (blue) and **6** (red). **1** shows the two peaks at 285.2 eV and 287.1 eV which represent the non–oxygenated C atom and oxygenated C atom, respectively. There are four kinds of carbon atoms in the different oxygen functional groups, C–OH (286.4 eV), C–O–C (287.2 eV), C=O (287.7 eV) and O=C–O (288.8 eV)[1]. For **4**, the peak at 286.8 eV broadens and the peak at 284.7 eV

sharpens compared to **1**. This result indicates that the oxygen functional groups are efficiently removed by the thermal reduction process.

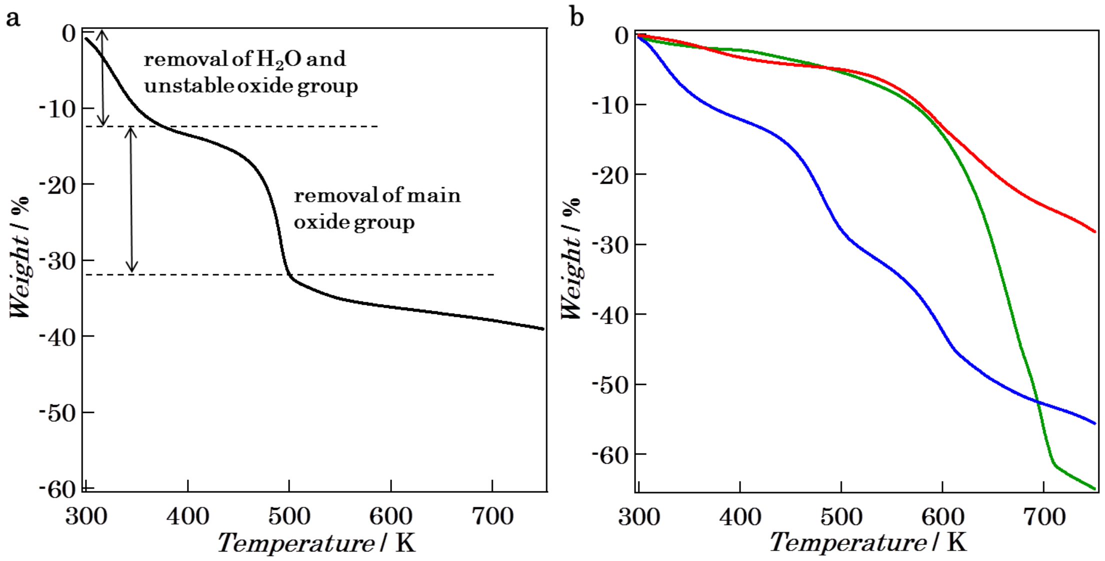

Supplementary Figure 5 Thermogravimetric curves for (a) pristine GO, (b) [Fe(Htrz)$_2$(trz)](BF$_4$) NPs (green), **1** (blue) and **4** (red). The [Fe(Htrz)$_2$(trz)](BF$_4$) NPs are stable up to 580 K and hence the [Fe(Htrz)$_2$(trz)](BF$_4$) NPs do not decompose during the thermal treatment at 473 K.

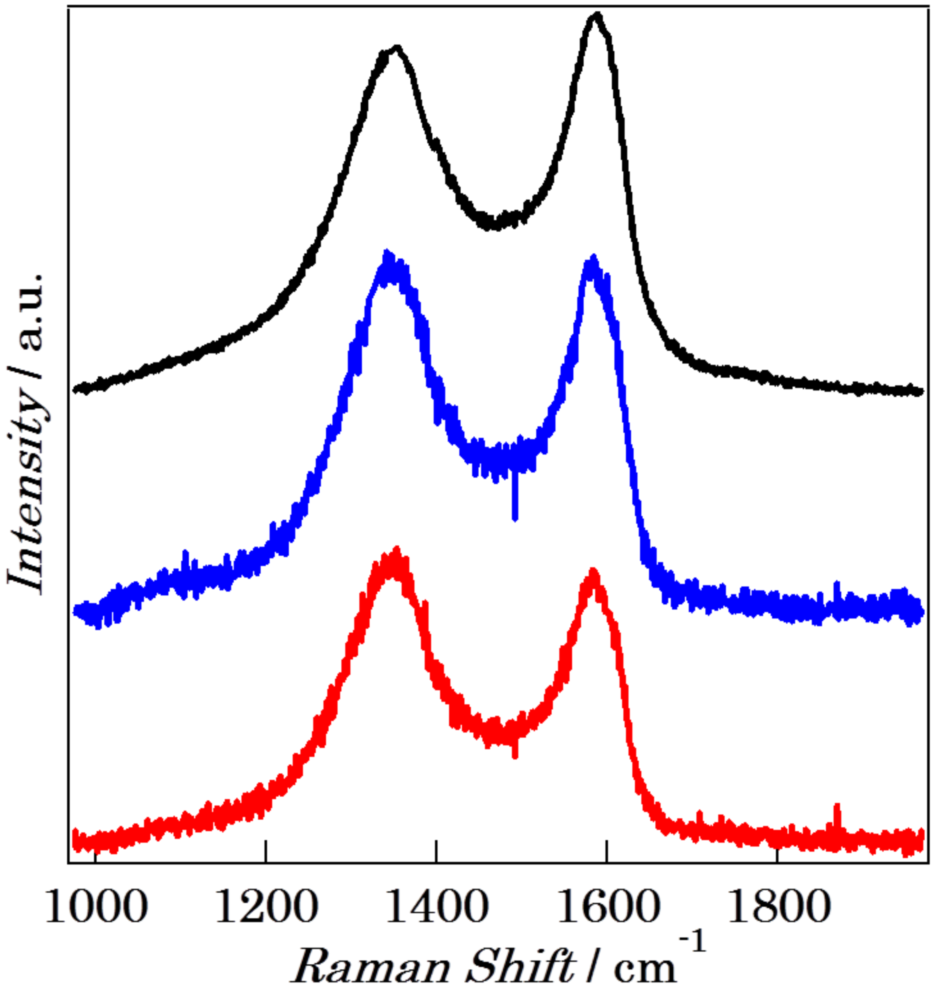

Supplementary Figure 6 Raman spectra of GO (black), **1** (blue) and **4** (red). The Raman spectra of **1** and **4** show typical bands for GO at 1356 cm^{-1} (D band) and 1585 cm^{-1} (G band), which are respectively the breathing mode of A_{1g} and the in-plane bond stretching mode of sp^2 C atoms (E_{2g})[2,3]. The peak ratio (I_D / I_G) for GO increases from 0.915 to 1.01 in **1** and to 1.08 in **4**. I_D / I_G is inversely proportional to the average size of the sp^2 domains[4]. As a consequence, sp^2 domains are created by reducing **1**, but the individual domain sizes are smaller than before the reduction.

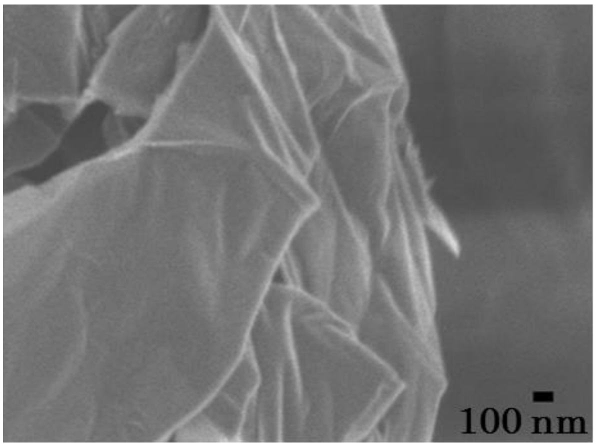

Supplementary Figure 7 SEM image of pristine GO.

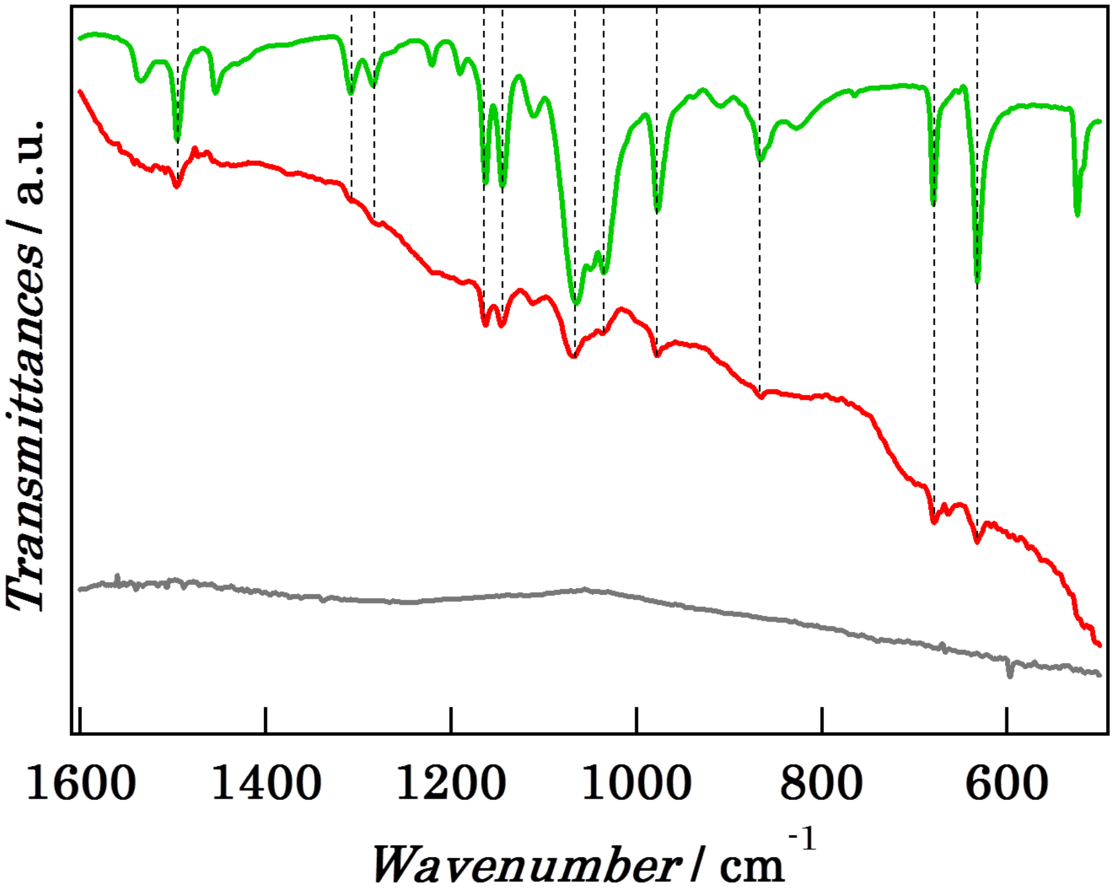

Supplementary Figure 8 FT–IR spectra for $[Fe(Htrz)_2(trz)](BF_4)$ (green), **4** (red) and pristine rGO (gray).

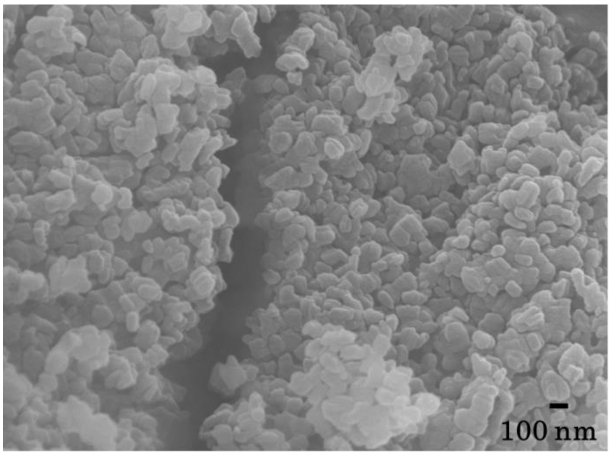

Supplementary Figure 9 SEM image of [Fe(Htrz)$_2$(trz)](BF$_4$) bulk particles.

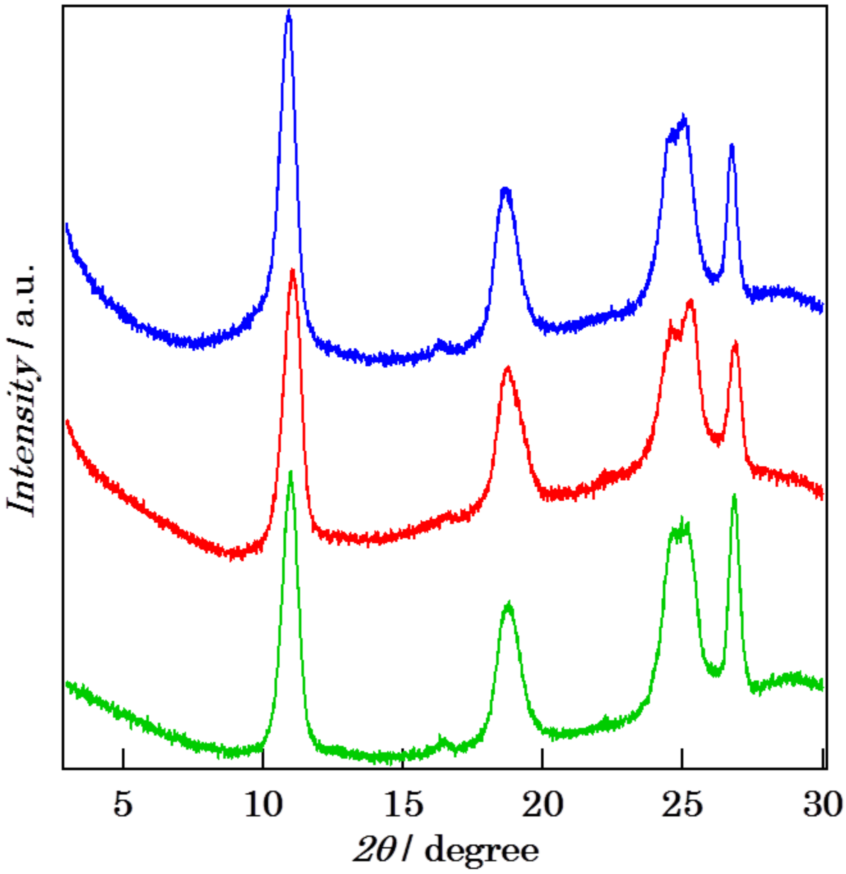

Supplementary Figure 10 PXRD patterns for bulk [Fe(Htrz)$_2$(trz)](BF$_4$) (green), **5** (blue) and **6** (red).

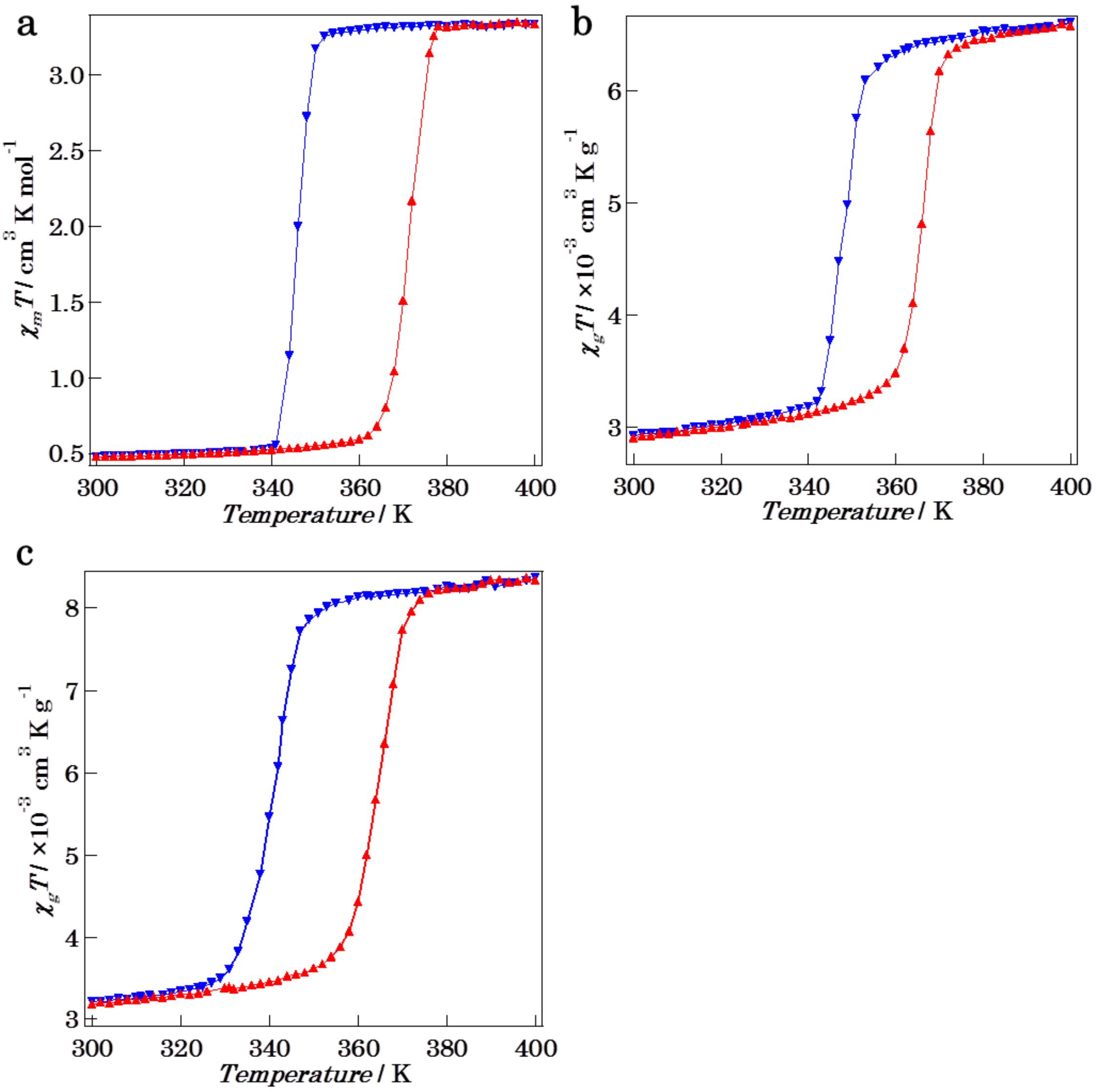

Supplementary Figure 11 SCO behavior of (a) bulk [Fe(Htrz)$_2$(trz)](BF$_4$), (b) **5** and (c) **6**; heating mode (▲) and cooling mode(▼).

SUPPLEMENTARY REFERENCES

1. Koinuma, M., Tateishi, H., Hatakeyama, K., Miyamoto, S., Ogata, C., Funatsu, A., Taniguchi, T. & Matsumoto, Y. Analysis of reduced graphene oxides by X-ray photoelectron spectroscopy and electrochemical capacitance. *Chem. Lett.* **42**, 924–926 (2013).
2. Tuinstra, F. & Koenig, J. L. Raman spectrum of graphite. *J. Chem. Phys.* **53**, 1126–1130 (1970).
3. Ferrari, A. C. & Robertson, J. Interpretation of Raman spectra of disordered and amorphous carbon. *Phys. Rev. B: Condensed Matter and Materials Physics* **61**, 14095–14107 (2000).
4. Stankovich, S., Dikin, D. A., Piner, R. D., Kohlhaas, K. A., Kleinhammes, A., Jia, Y., Wu, Y., Nguyen, S. T. & Ruoff, R. S. Synthesis of graphene–based nanosheets via chemical reduction of exfoliated graphite oxide. *Carbon* **45**, 1558–1565 (2007).
